# Global Warming Drives Phenological Shifts and Hinders Reproductive Success in a Temperate Octocoral

**DOI:** 10.1111/gcb.70660

**Published:** 2026-01-14

**Authors:** Núria Viladrich, Andrea Gori, Pol Capdevila, Maria Montseny, Andreu Santín, Ignasi Montero‐Serra, Marta Pagès‐Escolà, Joaquim Garrabou, Cristina Linares

**Affiliations:** ^1^ Departament de Biologia Evolutiva, Ecologia i Ciències Ambientals Universitat de Barcelona Barcelona Spain; ^2^ Institut de Recerca de Biodiversitat (IRBio) Barcelona Spain; ^3^ Institut de Ciències del Mar, Consejo Superior de Investigaciones Científicas Barcelona Spain; ^4^ CIIMAR–Interdisciplinary Centre of Marine and Environmental Research Matosinhos Portugal

**Keywords:** climate change, gorgonian, habitat‐forming species, marine, marine heatwaves, Mediterranean Sea, *Paramuricea clavata*, phenology

## Abstract

Global warming is profoundly reshaping biodiversity. Until now, most research has focused on the impacts of extreme temperature events. However, in many ecosystems, it is becoming increasingly apparent that climate change is accelerating the onset of spring warming conditions. These advanced warming conditions can significantly disrupt critical biological processes such as reproduction, which is key for population persistence. While interest in phenological shifts has increased in recent years, their effects on marine foundation species, such as corals, remain poorly understood. Here, we combined observational and experimental approaches to assess the effects of advanced spring warming conditions driven by climate change on the reproduction of the Mediterranean octocoral *Paramuricea clavata* , a foundation species. Our findings reveal that a 2°C warming leads to a 2‐week advancement in *P. clavata* spawning, as evidenced by both field observations, and *ex‐situ* experiments. These results underscore the role of advanced spring warming as a significant driver of phenological shifts in coastal marine ecosystems. Furthermore, we show that this phenological shift lead to a reduction in the number of spawning events, as well as decreases in larval biomass, survival rates, and settlement success. These findings highlight the urgent necessity to monitor phenological changes in foundational marine species, as such shifts can undermine the long‐term viability of coral populations and contribute to substantial decline in associated biodiversity. Consequently, the increased vulnerability of species caused by phenological responses driven by seasonal changes may lead to more dramatic consequences of ocean warming than previously anticipated.

## Graphical abstract

Climate change is advancing spring warming, yet its effects on marine foundation species remain unclear. We combined field observations and ex‐situ experiments to examine how advanced warming affects reproduction in the Mediterranean octocoral *Paramuricea clavata*. A 2°C increase triggered a 2‐week shift in spawning and reduced the number of spawning events. This phenological shift also decreased larval biomass, survival, and settlement success. Our results show that advanced spring warming is a major driver of phenological change and may threaten the long‐term viability of coral populations, potentially intensifying the ecosystem impacts of ocean warming.

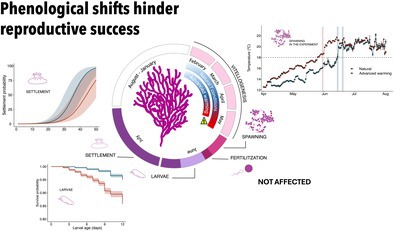

## 1 Introduction

Terrestrial, freshwater, and marine ecosystems have undergone a dramatic increase in the frequency and intensity of extreme thermal anomalies e.g., (Rogers and Dougherty 2019; Vitasse et al. 2022). The ecological consequences of these anomalies are inducing changes to all levels of biological organization, from genes to populations, communities, and ecosystems (e.g., Grimm et al. 2013; Liu et al. 2018; McCarty 2001; Scheffers et al. 2016). These environmental changes are reshaping earth's biodiversity, with major consequences for the services provided to human societies (Pecl et al. 2017). However, climate change goes beyond the occurrence of extreme thermal anomalies, and its biological consequences can be substantially more complex (e.g., Loeb et al. 1997; Parmesan 2006).

One aspect of climate change that remains understudied is the consequences of changes in seasonal temperature patterns. In many ecosystems, climate change is accelerating the onset of spring warming conditions (Walther et al. 2002). Since the warming‐up pattern during spring is one of the main drivers of phenological processes in terrestrial and marine species (e.g., growing seasons, migrations, or timing of reproduction), the advancement of the increase in temperature can significantly alter the timing of these processes resulting in ecological consequences at different levels (e.g., Ge et al. 2015; Greve et al. 2005; IPCC 2022; Parmesan 2006; Poloczanska et al. 2013; Thackeray et al. 2016). Due to the more widespread and persistent anomalous temperatures occurring in marine ecosystems compared to terrestrial ones (Fox‐Kemper et al. 2021), coupled with the fact that marine species are typically adapted to relatively stable temperatures (Pinsky et al. 2019; Pörtner 2021), phenological shifts might represent a greater threat to marine organisms than to their terrestrial counterparts (Burrows et al. 2011; Parmesan 2006; Poloczanska et al. 2013, 2016). Indeed, the phenology of marine species is changing at a faster rate (4.4 days per decade) than for terrestrial species (2.3–2.8 days per decade; Burrows et al. 2011; Parmesan 2006; Poloczanska et al. 2013, 2016). The ecological consequences driven by these phenological changes in marine species have resulted in disruptions in migratory patterns (Ganley et al. 2022; Robinson et al. 2009), breeding events (Neeman et al. 2015), as well as mismatched synchrony in species occurrence (Chivers et al. 2020; Edwards and Richardson 2004). However, little is known about the phenological impacts of ocean warming on marine foundation species such as seagrasses, macroalgae, or corals (but see, Peirano et al. 2011; Shlesinger and Loya 2019; Wahl et al. 2015). This knowledge gap is particularly alarming, as foundation species play a key structural role, and their degradation can have important consequences for biodiversity and ecosystem functioning (Bruno and Bertness 2001; Jones et al. 1997). The impacts of seasonal changes may be particularly severe in biodiversity hotspots, such as the Mediterranean Sea. Despite covering less than 1% of the Earth's ocean surface, the Mediterranean Sea presents a disproportionately high diversity of marine species, with up to 18% of the world's macroscopic marine species inhabiting its waters (Coll et al. 2010). Remarkably, 25 to 30% of these species are endemic to the region, highlighting the extraordinary biodiversity present despite its relatively small area (Bianchi and Morri 2000; Cuttelod et al. 2009). This high biodiversity sustains vital ecosystem functions and provides ecosystem services supporting an exceptional sociocultural and economic richness (Radhouane 2013; Randone et al. 2017; Tovar‐Sánchez et al. 2019). However, the Mediterranean Sea is highly vulnerable to the impacts of climate change (Cramer et al. 2018), placing its biodiversity and endemic marine species at significant risk of extinction due to limited opportunities for northward migration (Balzan et al. 2020; Kovats et al. 2014; Poloczanska et al. 2013). Warming in the Mediterranean Sea is occurring three to six times faster than the mean world's oceans (Pisano et al. 2020), making the Mediterranean Sea a prominent “climate change hotspot” (Cramer et al. 2018; Giorgi 2006).

In the Mediterranean Sea, about 50% of all recorded mass mortality events have occurred in Cnidarians, mainly octocorals (Garrabou et al. 2019, 2022). The high vulnerability of these species to changing temperatures may have dramatic consequences for biodiversity at community or ecosystem level, due to their paramount role as foundation species (Ballesteros 2006; Gili and Coma 1998). While most research has focused predominantly on the effects of summer heatwaves on octocoral survival, physiology and reproduction (e.g., Arizmendi‐Mejía et al. 2015; Ezzat et al. 2013; Kipson et al. 2012; Garrabou et al. 2019; Gómez‐Gras et al. 2021), the potential effects of advanced spring warming driven by sustained ocean warming on their reproductive phenology remain unknown. Indeed, although Shlesinger and Loya (2019) alerted that a dramatic shift in the timing and duration of reproductive events is occurring among Red Sea coral species, the evidence of climate change altering coral phenology is still scarce (but see Liberman et al. 2021; Shlesinger and Loya 2019).

Control of coral reproductive timing is complex and may involve an array of environmental signals driving gamete maturation and spawning (Baird et al. 2009). Environmental cues work at progressively finer scales to regulate the time of year, the days of spawning, and the time of spawning (Babcock et al. 1986). Traditionally, temperature has been considered the major seasonal cue (Baird et al. 2009). The increase in seawater temperature in spring, but also solar insolation and wind speed (van Woesik et al. 2006, 2009), induces gamete maturation determining spawning months (Fan and Dai 1999; Keith et al. 2016; Sakai et al. 2020). Once gametes have matured, corals become sensitive to moonlight and tide to synchronize their spawning days (Harrison et al. 1984; Lin et al. 2021; Komoto et al. 2023).

However, recent evidence also highlighted the role of seawater temperature in determining days and hours of coral spawning (Lin and Nozawa 2023). Climate change is consequently likely to significantly affect coral reproductive phenology and success (Baird et al. 2009).

Here, we aim to investigate the effects of advanced spring warming in the reproductive phenology and their consequences on fertilization, larval viability and biomass, as well as settlement rates in a foundation species of the Mediterranean benthic ecosystems (the octocoral *Paramuricea clavata* (Risso, 1826)). To achieve these objectives, we combined observational and experimental approaches. In the field, spawning events and seawater temperature were monitored over a 15‐year period (2005–2019). In the laboratory, advanced spring warming (as recorded in 2011 in the field) was experimentally replicated and the effects on the species' reproduction were assessed and compared with natural springs. We hypothesize that advanced reproductive events may force shorter gametogenic cycles and, consequently, reduce the size and quality of maturing gametes and the resulting larvae. Given that eggs are mainly composed of lipids (60%–70% dry weight, Arai et al. 1993), smaller eggs imply larvae with reduced energetic reserves to survive and disperse (Guizien et al. 2020), affecting local recruitment and genetic connectivity among populations (Cowen et al. 2000). It is extremely urgent to identify the changes induced by global warming on coral reproductive phenology and understand its impacts on all stages of the coral reproductive cycle (i.e., gametogenesis, spawning, larval survival, and settlement). This knowledge is crucial, as phenological shifts are already occurring in the field, with unknown consequences for the future viability of coral populations.

## 2 Material and Methods

### 2.1 Model Species

**FIGURE 1:**
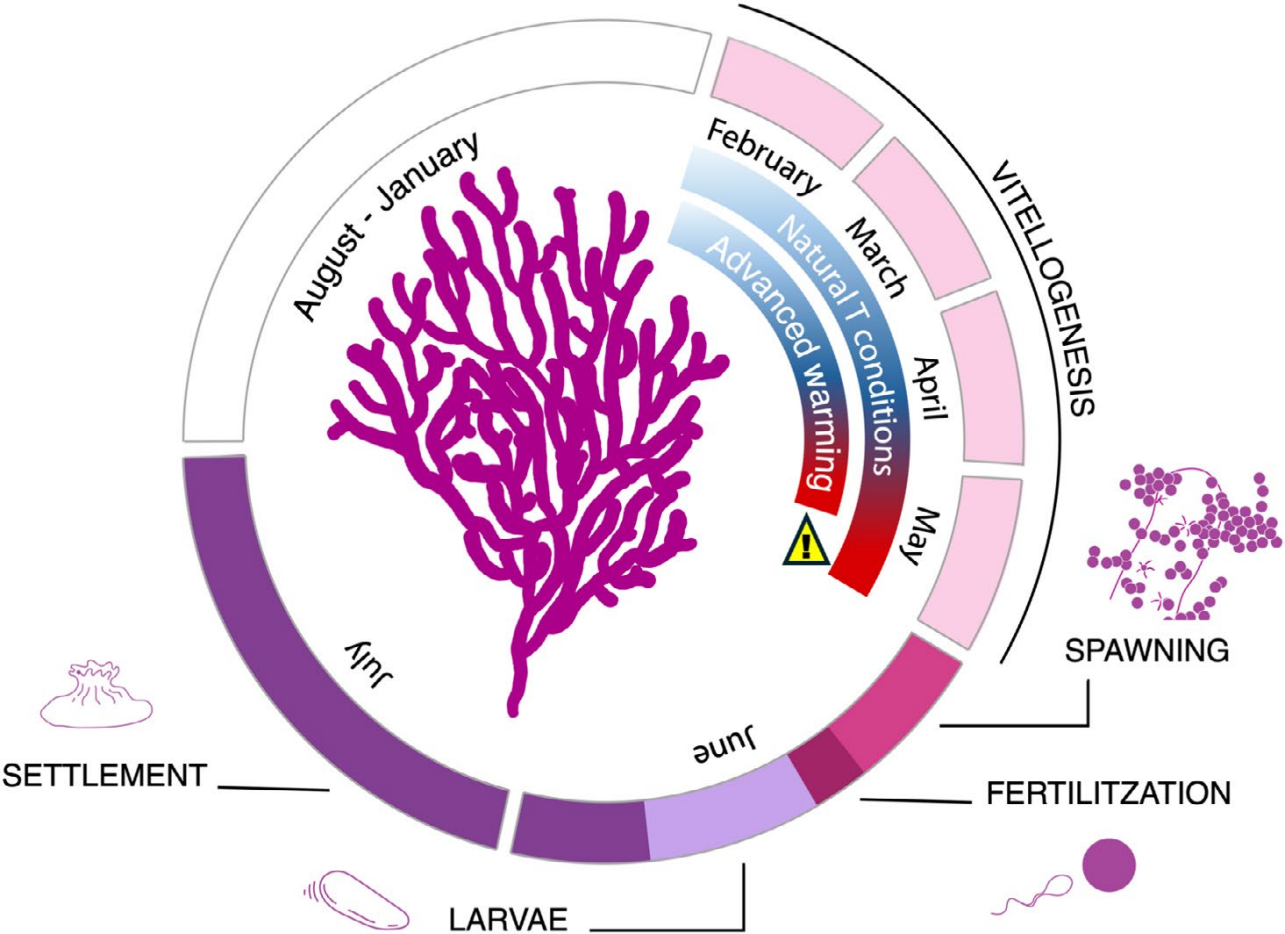
Reproductive cycles of the study species *Paramuricea clavata* . Timeline of key reproductive stages: Vitellogenesis (light pink), spawning (fuchsia), fertilization (dark fuchsia), larval pre‐competence (light violet), and settlement of competent larvae (violet). Data sourced from Coma, Ribes et al. (1995), Linares et al. (2008), and Zelli et al. (2020).

*Paramuricea clavata* is one of the major structuring species of Mediterranean coralligenous assemblages, supporting high biodiversity (Carpine and Grasshoff 1975; Ponti et al. 2018). It is a gonochoric surface brooder octocoral, and the development of sexual products lasts between 13–18 months for oocytes and 6–7 months for spermatic sacs (Coma, Ribes et al. 1995). However, the ripening and diameter of sexual products increase exponentially (vitellogenesis) during the last 4–5 months of development in both sexes (Figure 1). The spawning of *P. clavata* typically occurs in two distinct events in June, each lasting 2–3 days and separated by intervals of 7 to 13 days (Coma, Ribes et al. 1995; Linares et al. 2008). The eggs' fertilization occurs either immediately before eggs are released or on the colony surface shortly after release (Linares et al. 2008). Planulae typically appear after 48–72 h, with settlement starting 8–9 days after spawning (Linares et al. 2008; Zelli et al. 2020) (Figure 1).

### 2.2 Seawater Temperature and *P. clavata* Spawning at Field

Seawater temperature was recorded hourly from April to July between 2005 and 2019 at Pota del Llop in Medes Islands (42° 02′56″ N; 003°13′34″ E, northwestern Mediterranean Sea) with Hobo Pro V2 temperature data loggers located at 15 m depth obtained from the collaborative T‐MEDNet initiative (https://www.t‐mednet.org). During this period, the first spawning event of *P. clavata* populations in the Medes Islands was also recorded in most years.

### 2.3 Gorgonian Sampling and Experimental Set‐Up

Thirty colonies of *P. clavata* (15 female and 15 male colonies) were sampled at 15–20 m depth by SCUBA diving in February 2016, when sexual products begin their increase in size and maturation (vitellogenesis) (Coma, Ribes et al. 1995). Sampled colonies were higher than 30 cm (sexually mature, according to Coma, Zabala and Gili 1995), and their sex was already known from previous sampling (see details in Viladrich et al. 2016). After the sampling, the colonies were quickly transferred to the Experimental Aquarium Zone facilities at the Institute of Marine Sciences (ICM‐CSIC) in Barcelona (Spain). During transport (~3 h), each sampled colony was stored in plastic bags filled with seawater, all submerged in seawater at the environmental temperature recorded during the sampling (12°C), in a cooler plastic box. At the aquarium facilities, each colony was divided into two fragments to be distributed between the two experimental treatments (natural and advanced spring warming), thereby controlling for potential genetic effects. Five fragments from female and five from male colonies were located into each one of the six experimental tanks (50 L each, three replicates per treatment) (Figure S1). The temperature of each treatment was maintained with a titanium heater (Aqua Medic AM‐300) connected to an electronic controller (Aqua Medic TH‐100). For the “natural treatment,” seawater temperature was manually adjusted every few days to follow the natural cycle of temperature recorded from February to August at field. Conversely, in the “advanced warming treatment,” temperature was adjusted to follow the advanced rise recorded in 2011 at field (natural cycle +2°C) until reaching the maximum temperature of 20°C–22°C. Water in each tank was continuously renewed with a flow (60 L h^−1^) of Mediterranean seawater pumped from 15 m depth and filtered by 10 μm. A continuous water movement in each tank was provided by a submersible pump with a flow rate of 3200 L h^−1^, while water temperature was recorded by Hobo Pro V2 temperature data loggers. Gorgonians were kept under dim light conditions (~10–20 μmol photons m^−2^ s^−1^), as in their natural environment (Ballesteros 2006), on a light:dark regime daily adjusted to sunrise and sunset times, and fed five times a week with de‐frozen *Cyclops* (Ocean Nutrition, approximately 55 mg of dry weight per tank).

### 2.4 Larvae Collection

Once spawning was detected in a tank, eggs were collected from the surface of female branches and the tank bottom using 60 mL syringes, following established methods (e.g., Freire et al. 2019; Hata et al. 2017; Linares et al. 2008). Collected eggs were quickly cleared with abundant filtered seawater (0.2 μm) to eliminate mucous material. The eggs obtained from each tank and spawning event were maintained separately, and after ~72 h, when it is expected that fertilized eggs had transformed into larvae (Linares et al. 2008), the number of eggs non‐fertilized and transformed into larvae was counted. For each tank (*n* = 3) and treatments (*n* = 2), 300 of the obtained larvae were randomly transferred into three culture flasks with filtered seawater (10 μm) (300 larvae per container, 900 larvae per tank, 2700 larvae per treatment) (Figure S1). Culture flasks (750 mL) were made of porous plastic, which has been previously observed to be appropriate for settlement of *P. clavata* larvae (personal observation). Containers were maintained at 20°C–22°C (according to temperature recorded at field after spawning), and seawater completely renovated every 2 days. Since all flasks were prepared simultaneously and maintained under the same seawater supply and renewal regime, settlement cues, including culture flask material and biofilm, were strictly identical across treatments.

### 2.5 Larval Biomass

Larval biomass was quantified from triplicate GF/F filters. Each GF/F filter was pre‐combusted (5 h at 450°C) and loaded with exactly 30 larvae. All larvae were collected at the same developmental stage, immediately after planula formation (~72 h post‐spawning). Filters were immediately frozen in liquid nitrogen and stored at −80°C. Just before analysis, filters were acidified with HCl, dried at 60°C for 24 h, and analyzed for organic carbon quantification using a C/N autoanalyzer (Perkin‐Elmer 2040). The carbon content measured for each filter was normalized by the number of larvae (*n* = 30) and expressed as μg C per larva.

### 2.6 Larval Survival and Recruitment Rates

Larval survival was assessed every 2 days by counting the number of larvae in each container until day 12 after spawning. This period was selected to represent the pre‐competence period of *P. clavata* larvae, defined as the number of days between larval release and larval settlement (Linares et al. 2008; Zelli et al. 2020). Every 2 days, the settlement rate and post‐settlement survival were also assessed by counting the number of larvae settled in each container during 50 days from spawning. At the same time, and given that metamorphosis can occur without attachment to the substratum (Linares et al. 2008), the number of larvae pelagically metamorphosed into primary polyps and their survival was also assessed every 2 days during 50 days from spawning.

### 2.7 Statistical Analyses

All the statistical analyses were performed in R v4.0.0 (R Core Team 2021). Fertilization rates and larval biomass were compared among larvae collected from natural conditions and advanced treatment by *t*‐test. Before performing the *t*‐test, normality of data residuals and variance homogeneity were tested with Shapiro—Wilk and Bartlett tests, respectively (R—language functions “shapiro.test” and “bartlett.test”). Larval biomass graphs were performed using the “ggplot” package (Wickham 2016). To analyse the larvae survivorship, we used the Kaplan–Meier product‐limit method (Kaplan and Meier 1958), a non‐parametric statistic used to estimate the survival function from lifetime data. The larvae still alive after 12 days of the experiment represented censored data since they did not reach the outcome of interest during the study (i.e., death or settlement). In the analysis, a value of 0 was assigned to these incomplete observations, whereas a value of 1 was assigned to complete observations (i.e., at time points of larval death or settlement during the study). As the Kaplan–Meier method does not allow for incorporation of replicate information into the analysis, the analysis was performed by pooling data from all flasks together for each treatment. Pooling yields a single, straightforward estimate of the treatment survival curve but will underrepresent the uncertainty coming from between‐flask variation. For completeness, we therefore also provide Kaplan–Meier curves for each flask separately. To test the differences in larval survival among treatments, the log‐rank test was used. This non‐parametric test compares the survival distributions of the two treatments assuming the null hypothesis (no difference in survival) and is based on the rank ordering of survival times that can be applied to censored data (Mantel 1966; Peto and Peto 1972). Larval biomass graphs were performed using the “ggplot2” package (Wickham 2016).

To analyze the settlement and metamorphosis probability of larvae (response variables) we used multilevel Bayesian models (McElreath 2020). We fitted two separate models, where the number of larvae settled and metamorphosed is assumed to follow a binomial distribution with parameter *n* (the total number of trials) and probability of success (*p*). The linear predictor for the probability of settlement and metamorphosis included an interaction term between days and treatment as fixed effects, as well as a varying intercept for each replicate as a random effect. The model also included a first‐order autoregressive (AR(1)) process within each replicate to model temporal correlations in the residuals over time.

The structure of the models was:

(1) $$Y_{i}\sim \text{Binomial}\left(n_{i},p_{i}\right)$$

(2) $$\begin{array}{c} \text{logit}\left(p_{i}\right)=\alpha +\alpha_{j\left[i\right]}+\beta_{1}{\text{Year}}_{\left[i\right]}+\beta_{2}{\text{Treatment}}_{\left[i\right]}\\ +\beta_{3}\left({\text{Year}}_{\left[i\right]}\times {\text{Treatment}}_{\left[i\right]}\right)+\varepsilon_{i} \end{array}$$

(3) $$\varepsilon_{i}\sim \mathrm{AR}\left(1,\Phi \right)$$

We used weakly informed priors:

(4) $$\alpha \sim \text{Normal}\left(0,1\right)$$

(5) $$\alpha_{j}\sim \text{Normal}\left(\bar{\alpha },\sigma_{\alpha }\right)$$

(6) $$\bar{\alpha }\sim \text{Normal}\left(0,1\right)$$

(7) $$\beta_{1}\text{Year}\sim \text{Normal}\left(0,1\right)$$

(8) $$\beta_{2}\text{Treatment}\sim \text{Normal}\left(0,1\right)$$

(9) $$\sigma_{\alpha }\sim \text{Exponential}\left(1\right)$$

(10) $$\mathrm{AR}\sim \text{Beta}\left(2,2\right)$$

(11) $$\Phi \sim \text{Student}\;T\left(3,0,0.25\right)$$

where $Y_{i}$ represents the settlement and metamorphosed larvae of the *j*th replicated and is given by a Binomial distribution were the parameter p represents the probability of settlement or metamorphosis. α represents the intercept and $\alpha_{j}$ is the independent random intercept for each replicate (*j*). $\beta_{1}\text{Year}$ is the effect of the continuous factor Year. $\beta_{2}\text{Treatment}$ is the effect of the discreate factor Treatment. AR(1) represents the autoregressive process of order 1.

Similarly, to analyse the settlement and metamorphosis of larvae (response variables) we also used two multilevel Bayesian models, one with the number of settled and another with the number of metamorphosed larvae as response variables. The fixed effects of both models were larval age (continuous) and treatment conditions (discrete). We considered each replicate as the random intercept. To account for the temporal non‐independence of the total area and necrosis area data, we included an autoregressive‐1 temporal process, which assumes neighboring observations within the time series to be more similar than further ones.

The structure of the models was:

(12) $$Y_{i}\sim \text{Poisson}\left(\lambda_{i}\right)$$

(13) $$\begin{array}{c} \mathrm{Log}\left(\lambda_{i}\right)=\alpha +\alpha_{j\left[i\right]}+\beta_{1}{\text{Year}}_{\left[i\right]}+\beta_{2}{\text{Treatment}}_{\left[i\right]}\\ +\beta_{3}\left({\text{Year}}_{\left[i\right]}\times {\text{Treatment}}_{\left[i\right]}\right)+\varepsilon_{i} \end{array}$$

(14) $$\varepsilon_{i}\sim \mathrm{AR}\left(1,\Phi \right)$$

We used weakly informed priors:

(15) $$\alpha \sim \text{Normal}\left(0,1\right)$$

(16) $$\alpha_{j}\sim \text{Normal}\left(\bar{\alpha },\sigma_{\alpha }\right)$$

(17) $$\bar{\alpha }\sim \text{Normal}\left(0,1\right)$$

(18) $$\beta_{1}\text{Year}\sim \text{Normal}\left(0,1\right)$$

(19) $$\beta_{2}\text{Treatment}\sim \text{Normal}\left(0,1\right)$$

(20) $$\sigma_{\alpha }\sim \text{Exponential}\left(1\right)$$

(21) $$\mathrm{AR}\sim \text{Beta}\left(2,2\right)$$

(22) $$\Phi \sim \text{Student}\;T\left(3,0,0.25\right)$$

where $Y_{i}$ represents the settlement abundance and metamorphosed larvae of the *j*th replicated and is given by a Poisson distribution were the parameter λ represents the expected value of the settlement abundance or metamorphosed larvae. α represents the intercept and $\alpha_{j}$ is the independent random intercept for each replicate (*j*). $\beta_{1}\text{Year}$ is the effect of the continuous factor Year. $\beta_{2}\text{Treatment}$ is the effect of the discreate factor *Treatment*. AR(1) represents the autoregressive process of order 1.

All models were fitted using the “brms” package v2.1.0 (Bürkner 2017). Models were run for 8000 iterations, with a warmup of 800 iterations. To check the validity of our multilevel Bayesian models we ran a set of diagnostics. We inspected model convergence by visually examining trace plots and using “Rhat” values (the ratio of the effective sample size to the overall number of iterations, with values close to one indicating convergence). We also evaluated the model fit by exploring the distribution of the residuals, their variance, and the posterior predictive checks (Figures S2–S4). All the diagnostics suggested a good model fit.

## 3 Results and Discussion

### 3.1 Shifts in Reproductive Phenology of the Mediterranean Octocoral *Paramuricea clavata* in Response to Ocean Warming

**FIGURE 2:**
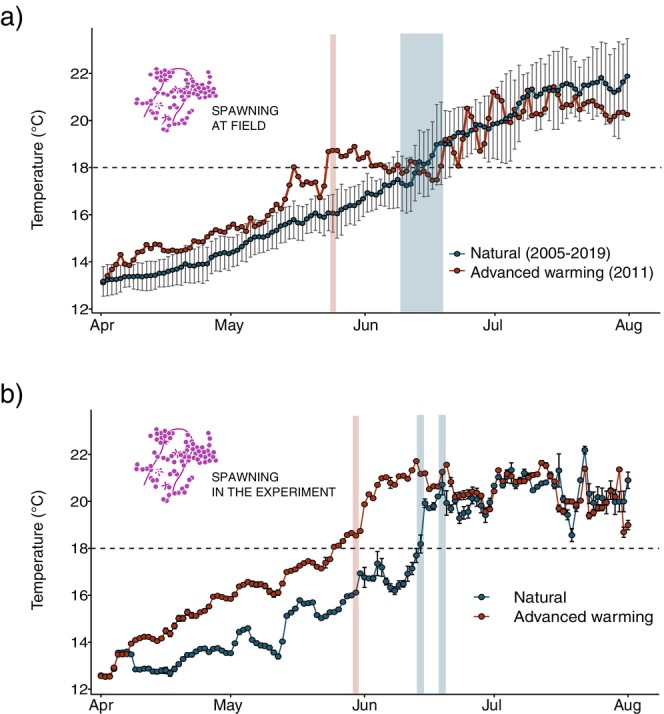
(a) Spring increase in seawater temperature recorded at sea. Mean (±SD) temperatures recorded at 15 m depth (Pota del Llop, Medes Islands) between 2005 and 2019 (blue), and temperatures in 2011 (red). Data from the collaborative T‐MEDNet initiative (www.t‐mednet.org). Light bars indicate first spawning event recorded in natural springs (blue) and in 2011 (red). (b) Seawater temperature recorded in the experiment. Natural (blue) and advanced warming (red) treatments. Light bars indicate the spawning events in natural (blue) and advanced warming (red) treatments.

In the present study, we revealed that in 2011, the Mediterranean octocoral *Paramuricea clavata* experienced a significant shift in its reproductive phenology (Figures 1 and 2). From 2005 to 2019, the first spawning event consistently occurred between the 9th and the 18th of June (within 7 days around full or new moon), coinciding with annual water temperature increases to 18.0°C–18.5°C, as previously reported (Coma, Ribes et al. 1995; Linares et al. 2008). However, in 2011, unexpected spawning of *P. clavata* colonies was observed on the 24th of May (7 days after full moon) at depths between 15 and 35 m (Figure 2a). This unusually advanced spawning event occurred when the local seawater temperature at 15 m depth was 18.3°C, the warmest May temperature recorded in the last 20 years in the study area (Figure 2a).

In marine pelagic species, most of the reported advanced phenological responses have been attributed to ocean warming (Pörtner et al. 2022). However, in coastal systems, the observed shifts in species phenology are especially difficult to attribute solely to ocean warming, since human impacts are pronounced and ubiquitous (Allan et al. 2023; Williams et al. 2022). Indeed, Shlesinger and Loya (2019) reported that several plausible drivers may contribute to the observed changes in spawning timing in Red Sea coral species, including human polluting activities and climate change. However, our results, combining long‐term monitoring, observational data, and laboratory experiments, clearly showed that a 2°C faster warming in spring triggered a 2‐week advancement in *P. clavata* spawning. Indeed, in controlled aquaria experiments, the spawning of colonies maintained under natural conditions started on the 14th of June (6 days before full moon) (consistent with field observations) at temperatures between 17.8°C and 18.2°C, whereas in the advanced warming treatment, spawning occurred 2 weeks earlier (5 days before new moon) at temperatures between 18.5°C and 18.7°C (Figure 2b). Warmer seawater temperatures also advanced spawning in tropical corals (Komoto et al. 2022; Nozawa 2012; Sakai et al. 2020), and transplants in reciprocal transplantations between sites differing in thermal regimes spawned earlier at the warmer sites (Fan and Dai 1999; Lin and Nozawa 2023). Spring increase of temperature may act as a proximate environmental cue for spawning months (Keith et al. 2016), promoting the maturation of oocytes at the late oogenesis (Nozawa 2012; Shlesinger et al. 1998). Indeed, an effect of seawater temperature on the gametogenesis period and larval release timing has also been reported in brooding corals (Crowder et al. 2014; Fan et al. 2017; McRae et al. 2021). Since this regulation of coral spawning by temperature (Lin and Nozawa 2023), global reproductive phenological shifts in corals is to be expected as a consequence of global warming worldwide. The still limited detection of such reproductive phenological changes could be partially caused by the fact that much data on coral sexual reproductive patterns is hardly published (Baird et al. 2021), as well as due to a general lack of long‐term data on the timing and duration of spawning events for most coral species (Lin and Nozawa 2023; Viladrich, Bramanti, et al. 2022). However, as global warming accelerates and thermal variability increases, it becomes crucial to monitor phenological shifts in foundation species like corals to accurately predict its consequences.

### 3.2 Consequences of Phenological Shift on Reproductive Success

It is widely accepted that phenological plasticity enables organisms to adaptively adjust their life cycle to changing environmental conditions. However, in the face of sustained directional environmental change (e.g., global warming), understanding the underlying mechanisms and effects of these phenological changes is key, as species persistence may depend on the degree of transgenerational impacts (Fox et al. 2019; Gaitán‐Espitia and Hobday 2021). In this study, we observed that advanced spring warming in *P. clavata* affects spawning, larval biomass, survival, and settlement rates. All these changes may severely undermine the long‐term viability of its populations (Linares et al. 2007).

It has been well documented that *P. clavata* spawning typically occurs in two discrete events separated by 7 to 13 days (Coma, Ribes et al. 1995; Linares et al. 2008). Indeed, under natural conditions, these events were observed on June 11 and 18, with a 7‐day interval of inactivity. However, under advanced warming conditions, the second spawning event was not observed in 2011 (Figure 2b). Although the causes are unknown, the reduction from two to one discrete spawning event observed under advanced treatment could decrease the chances of spawning occurring under optimal conditions for fertilization and, consequently, reduce the reproductive outcome (Lasker and Kim 1996; Penland et al. 2004; Stobart et al. 1992). Furthermore, coral populations limited to a single annual spawning event may experience reduced interbreeding opportunities among colonies, potentially leading to reproductive isolation, genetic divergence, and decreased genetic diversity over time (Gilmour et al. 2016).

**FIGURE 3:**
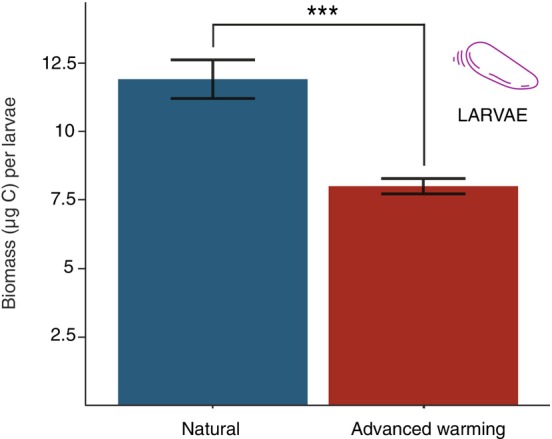
Larval biomass. Content of carbon (μg C per larvae) in the larvae from the natural (blue) and the advanced warming (red) treatments. Error bars are standard deviation (*n* = 3 replicates of 30 larvae per treatment). Significant differences are indicated by *** for *p* < 0.001.

**FIGURE 4:**
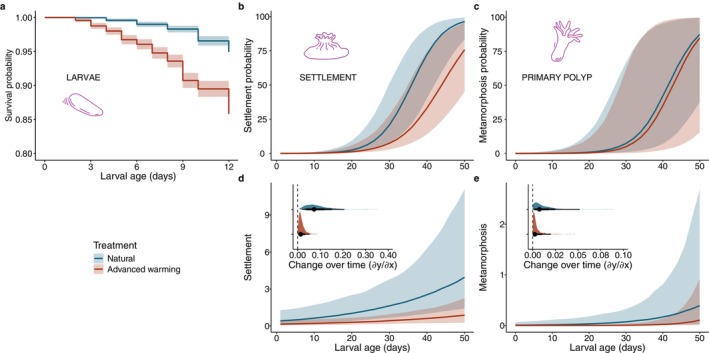
Advanced warming impacts key stages of *Paramuricea clavata* reproductive cycle. (a) Kaplan–Meier estimated larval survival probabilities, (b) settlement probability and (c) metamorphosis as a function of larval age, (d) estimated number of settlements and (e) estimated number of primary polyps from the metamorphosis of the larvae in the natural (blue line) and advanced warming (red line) treatments. Insets in (c, d) represent the settlement and metamorphosis rate over time for the natural (blue) and advanced warming (red) conditions. Shading represents 95% confidence intervals in (a) and the credible intervals in (b–e). For the absolute number of larvae, settlers and metamorphosed larvae refer to Tables S5–S7.

Our findings also point out that an advancement of reproductive phenology results in a reduced size of the produced larvae probably because of the shorter oogenesis. The increased metabolic rates associated with rising temperatures (Coma et al. 2000; del Alcázar‐Julià et al. 2025; Previati et al. 2010) may also diminish the energy allocated to reproduction, consequently affecting larval size. Additionally, late‐stage oogenesis in octocorals is characterized by rapid lipid accumulation (Baptista et al. 2012; Lin et al. 2012). Advanced warming temperatures may disrupt this process by accelerating metabolic turnover, potentially forcing colonies to complete vitellogenesis more rapidly or under energetic stress (Liu et al. 2024; Waller et al. 2023). This energetic stress can lead to incomplete yolk provisioning, producing oocytes with lower lipid reserves despite only moderate compression of the reproductive cycle (Lin et al. 2012; Viladrich et al. 2025). Since coral larvae are critically dependent on maternally provisioned lipids for development and survival (Arai et al. 1993; Figueiredo et al. 2012), maternal energetic deficits during oogenesis manifest as reduced larval biomass and quality (Padilla‐Gamiño et al. 2012; Viladrich et al. 2025). Thus, reduced larval biomass under advanced treatment may be likely results from both shortened gametogenesis and altered maternal energetic balance. Specifically, larval biomass immediately after spawning was significantly lower under advanced warming conditions, with a biomass of 8.0 ± 0.3 μg C per larva compared to 11.9 ± 0.7 μg C per larva under natural conditions (Figure 3; *t*‐test, *p* < 0.001). This reduction in larval biomass may explain the lower larval survival (Kaplan–Meier survival analysis, log‐rank test, *p* < 0.001, Figure 4a) and the lower settlement rates observed in the larvae produced by colonies exposed to the advanced warming compared to natural conditions (Figure 4b,d, Tables S1 and S2). Survival of larvae produced by colonies exposed to natural conditions remained above 95% during the pre‐competence period, whereas larvae produced by colonies maintained under advanced warming conditions showed a continuous decrease in survival from the outset, with a reduction of approximately 15% (Figure 4a). We must note, however, that the survival curves showed high variability among replicates (Figure S5). Although larvae produced by colonies maintained under both treatments started settling just after spawning, larvae produced by colonies maintained under natural conditions showed a gradual increase in settlement, reaching 4.3% ± 0.9% of settled larvae by day 22 post‐spawning (mean ± SE). In contrast, larvae produced by colonies exposed to an advanced warming showed consistently low settlement rates throughout the experiment, with a maximum settlement rate of only 0.9% ± 0.2%.

Across marine invertebrates, egg biomass is positively correlated with maternal investment per offspring (Emlet 1987; Jaeckle 1995; McEdward and Carson 1987). Following foundational theory by Vance (1973), the primary role of maternal investment is to provide offspring with the necessary resources to reach a stage where they can feed for themselves. Consequently, a shorter oogenesis may result in smaller eggs with lower energy reserves transferred by the mother colonies (Viladrich, Linares, and Padilla‐Gamiño 2022), reducing offspring survival or limiting their development. In our study, the observed failure in settlement of larvae produced by colonies exposed to the advanced warming conditions may suggest a diminished capacity to undergo metamorphosis. This could be also supported by the residual proportion of larvae metamorphosed without settling (15 out of 2700 larvae, 0.5%) observed only among those produced by colonies exposed to the advanced warming (Figure 4c,e, Tables S3 and S4). Larvae produced by colonies maintained under natural conditions started to metamorphose on day 12 and their proportion increased with time, reaching 1.3% ± 0.7% on day 50 (mean ± SE). In contrast, larvae produced by colonies in the advanced warming treatment did not start to metamorphose until day 30 and their proportion was residual throughout the study, with only 0.2% ± 0.1% on day 50. These results may be likely due to insufficient energy reserves in the eggs, since if larval energy levels drop below a critical threshold, settlement and metamorphosis may be compromised (Kempf 1981; Lucas et al. 1979; Richmond 1987). Indeed, experimental evidence shows that lipid‐rich larvae exhibit substantially higher settlement success than energetically depleted counterparts, indicating that larval energy reserves play a critical role in settlement and post‐settlement survival (Boulotte et al. 2023). Energetically compromised larvae may also display altered physiology, impaired competence, and reduced sensitivity to settlement stimuli, ultimately affecting settlement and metamorphosis success (Graham 2012; Jiang et al. 2017; Nozawa and Harrison 2000; Putnam et al. 2008). Since settlement cues were identical across treatments, the delayed metamorphosis likely reflects a physiological consequence rather than differences in external cues. However, although markedly lower settlement and metamorphosis rates under advanced warming likely reflect a biologically meaningful effect of reduced larval energetic reserves, the low settlement rates observed in our experiment make it difficult to draw accurate and generalizable conclusions. Further studies with larger sample sizes are needed to confirm these patterns and clarify the ecological consequences of phenological shifts in settlement dynamics.

The observations of phenological shift in coral reproduction already warned of a decline in new recruits (Shlesinger and Loya 2019). The authors attributed this recruitment failure to a reduced fertilization rate resulting from an extension in breeding periods leading to a breakdown in spawning synchrony among colonies of broadcast‐spawning coral species. Conversely, in our study, the advanced spawning under warming conditions in the surface‐brooder *P. clavata* is not affecting fertilization rates, being 91.3% in natural conditions and 92.0% under advanced warming treatment (*t*‐test, *p* = 0.86). Although obtained after maintaining parental colonies in experimental tanks, thus possibly facilitating fertilization, this finding is consistent with previous results from the surface‐brooding coral *Rhytisma fulvum*, which also maintained fertilization success under increased temperature and acidification (Liberman et al. 2021). These results suggest that phenological shifts may impact different ways depending on the coral reproductive strategy (i.e., broadcast, surface or internal brooder). Indeed, in broadcast‐spawning species, where eggs are externally fertilized, spawning synchronicity is crucial to ensure fertilization success (Alino and Coll 1989). Conversely, in surface‐brooder species, eggs are fertilized while they are retained by mucous material on the surface of female colonies, thereby increasing fertilization success despite spawning asynchrony (Kahng et al. 2011). However, the ultimate responses of coral species to global change could be conditioned by the rate and magnitude of environmental change, the organisms' capacity for acclimation, the degree of local adaptation of natural populations and their potential for adaptive evolution (Calosi et al. 2017; Pespeni et al. 2013; Vargas et al. 2017).

### 3.3 Ecological Consequences of Phenological Reproductive Shifts

Whether or not adaptive mechanisms exist, phenological responses to warming, together with the recurrent mass mortality events affecting foundational species, may increase the ecological vulnerability of benthic communities and contribute to conditions that favor ecosystem collapses or regime shifts (Canadell and Jackson 2021; Collins et al. 2019; Ma et al. 2021). Indeed, such regime shifts are increasingly reported not only in tropical coral reefs worldwide (e.g., Bell et al. 2021; Crisp et al. 2022; Jouffray et al. 2015; Mumby 2009), but also in the Mediterranean coralligenous community (Garrabou et al. 2021; Gómez‐Gras et al. 2021; Verdura et al. 2019).

The recruitment failure documented for *P. clavata* highlights potential risks for the long‐term persistence of this species at local and regional scales. The *P. clavata* species is also a charismatic and structurally important species within Mediterranean coralligenous assemblages, enhancing habitat complexity and contributing to both the ecological functioning and the aesthetic value of the underwater landscape (Ballesteros 2006; Harmelin and Marinopoulos 1994). Consequently, declines in its populations may indirectly affect the provision of marine ecosystem services, with potential socio‐economic implications (Liquete et al. 2016; Paoli et al. 2017). Moreover, although phenological responses to warming, and their consequences on reproductive output and early life‐history stages, may differ among species, populations, and environmental contexts, widespread reproductive disruption in foundation species may contribute to biodiversity loss within the Mediterranean Sea, a recognized hotspot of marine diversity (Bianchi and Morri 2000; Cuttelod et al. 2009). However, future research at larger spatial scales including more species with different reproductive strategies, also incorporating interannual environmental variability, will be essential to determine the extent to which the reproductive impairments observed here may be generalized across benthic communities.

Finally, there is no doubt that climate change is and will continue to be an important driver of species extirpations and extinctions (Briggs 2017; Cowie et al. 2022; IPCC 2022). To date, assessments of extinction risk have largely focused on predicted population abundance, physiology, and species distribution (Manes et al. 2021). However, the present results may suggest that the sublethal effects of phenological responses might have been overlooked, potentially increasing the number of species at high or very high risk of extinction.

## Author Contributions

**Núria Viladrich:** conceptualization, data curation, formal analysis, investigation, methodology, writing – original draft. **Andrea Gori:** conceptualization, investigation, methodology, writing – review and editing. **Pol Capdevila:** formal analysis, writing – review and editing. **Maria Montseny:** investigation, writing – review and editing. **Andreu Santín:** investigation, writing – review and editing. **Ignasi Montero‐Serra:** investigation, writing – review and editing. **Marta Pagès‐Escolà:** investigation, writing – review and editing. **Joaquim Garrabou:** funding acquisition, writing – review and editing. **Cristina Linares:** conceptualization, funding acquisition, investigation, writing – review and editing.

## Funding

This research has been partially supported by the CORFUN project (TED2021‐131622BI00) funded by MICIU/AEI and the EU NextGeneration, and the UndResCoral Project (PID2022‐137539OB‐C21) funded by MICIU/AEI and by FEDER, UE. N.V. was supported by a Beatriu de Pinós Fellowship (2022BP‐00097) from the Generalitat de Catalunya. P.C. was supported by Beca Leonardo a Investigadores y Creadores Culturales 2024 de la Fundación BBVA.

## Conflicts of Interest

The authors declare no conflicts of interest.

## Supporting information


**Appendix S1:** gcb70660‐sup‐0001‐Supinfo.pdf.

## Data Availability

The data that support the findings of this study, as well as the model used for the analyses, are openly available in the Zenodo repository through the link https://doi.org/10.5281/zenodo.17761932.
