# Supplementary material for: Global Warming Drives Phenological Shifts and Hinders Reproductive Success in a Temperate Octocoral
Source: Glob Chang Biol. 2026 Jan 14;32(1):e70660. doi: 10.1111/gcb.70660 (PMC12802391; doi:10.1111/gcb.70660)
Supplement: Supplementary file 1 — Appendix S1: gcb70660‐sup‐0001‐Supinfo.pdf. [file GCB-32-e70660-s001.pdf]

Supplementary Information

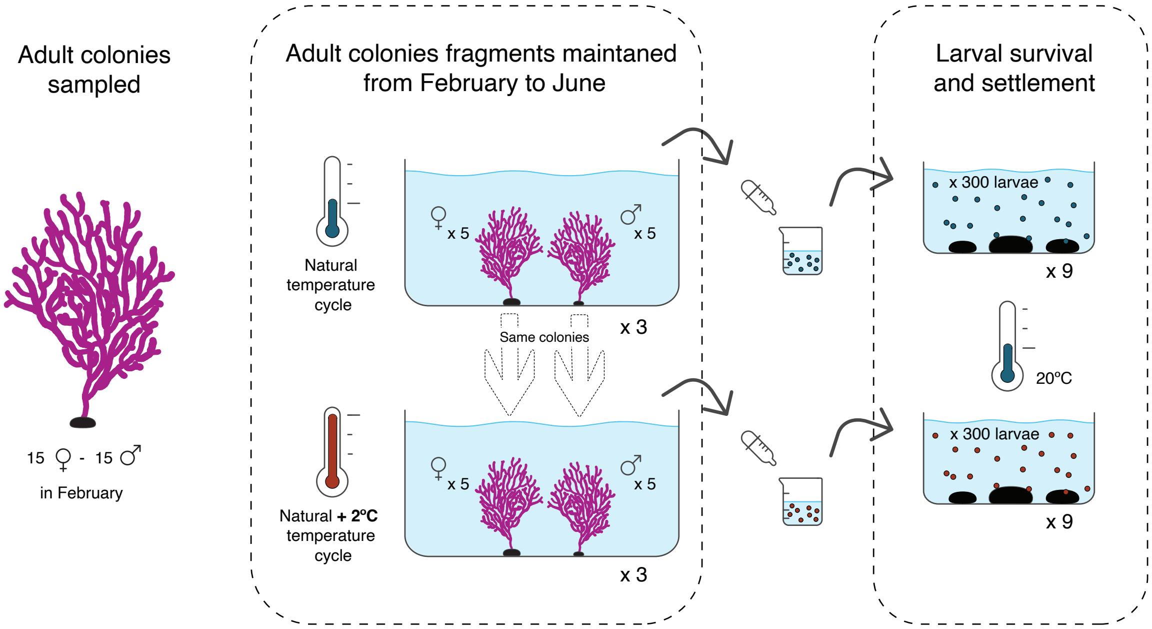

Figure S1 – The experimental design illustration showing the colonies sampled in February (both male and female), the number of fragments maintained under the experimental treatments (natural temperature and natural +2 °C temperature cycle), larval sampling, and the larvae maintained for assessing larval survival and settlement.

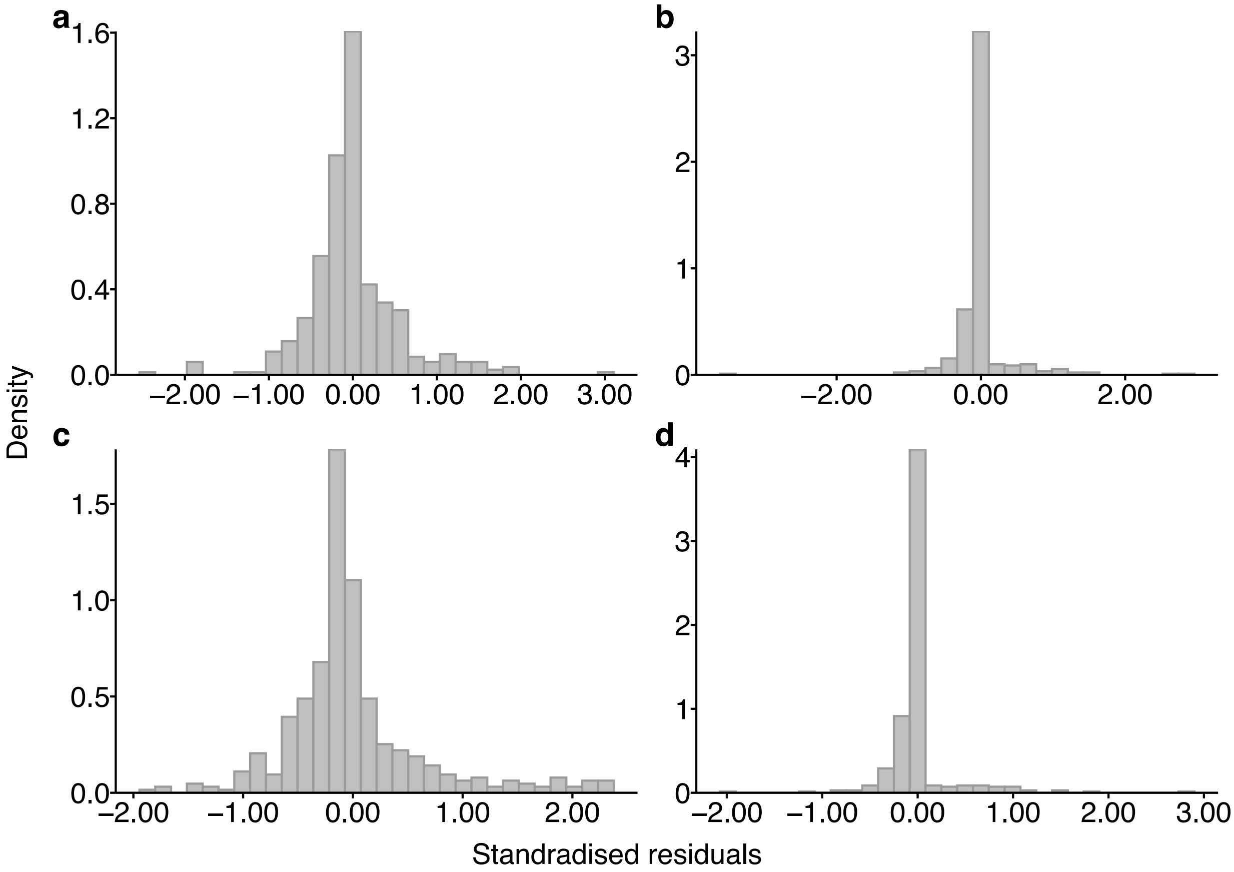

Figure S2 – Distribution of the standardised residuals for the multilevel Bayesian models Residuals were examined for the (a) settlement probability, (b) metabolism probability, (c). settlement rate and (d) metabolism rate.

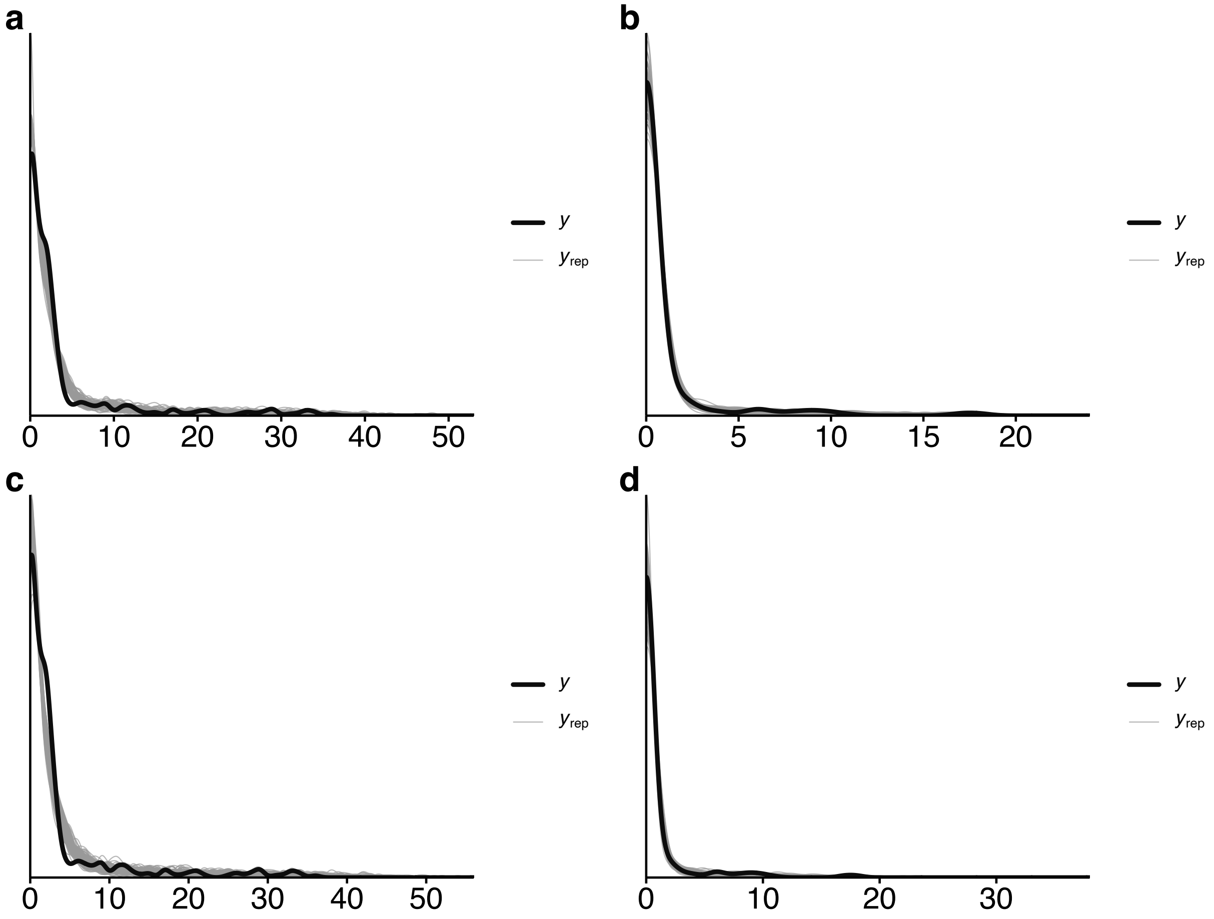

Figure S3 – The posterior predictive checks for the multilevel Bayesian models. The posterior predictive checks do not show strong discrepancies between our data (dark lines, y) from the predictions from the model (light grey lines, yrep) for any of the models. The posterior predictive checks were examined for the (a) settlement probability, (b) metabolism probability, (c) settlement rate and (d) metabolism rate.

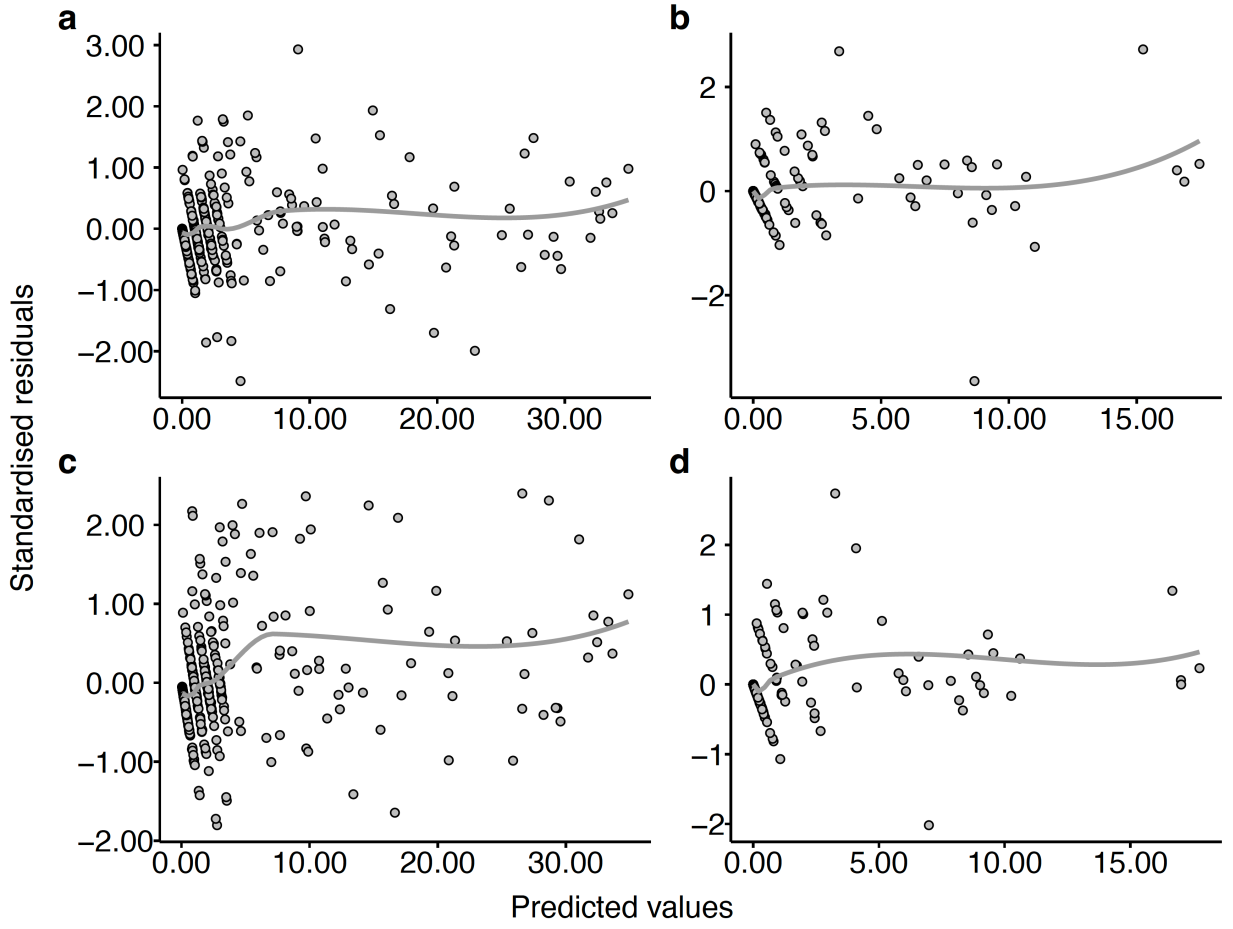

Figure S4 – The standardised residuals vs the predicted values for the multilevel Bayesian models. The standardised residuals vs the predicted values have similar variances for all models, suggesting that the equal variance assumption is met. Residuals were examined for the (a) settlement probability, (b) metabolism probability, (c) settlement rate and (d) metabolism rate.

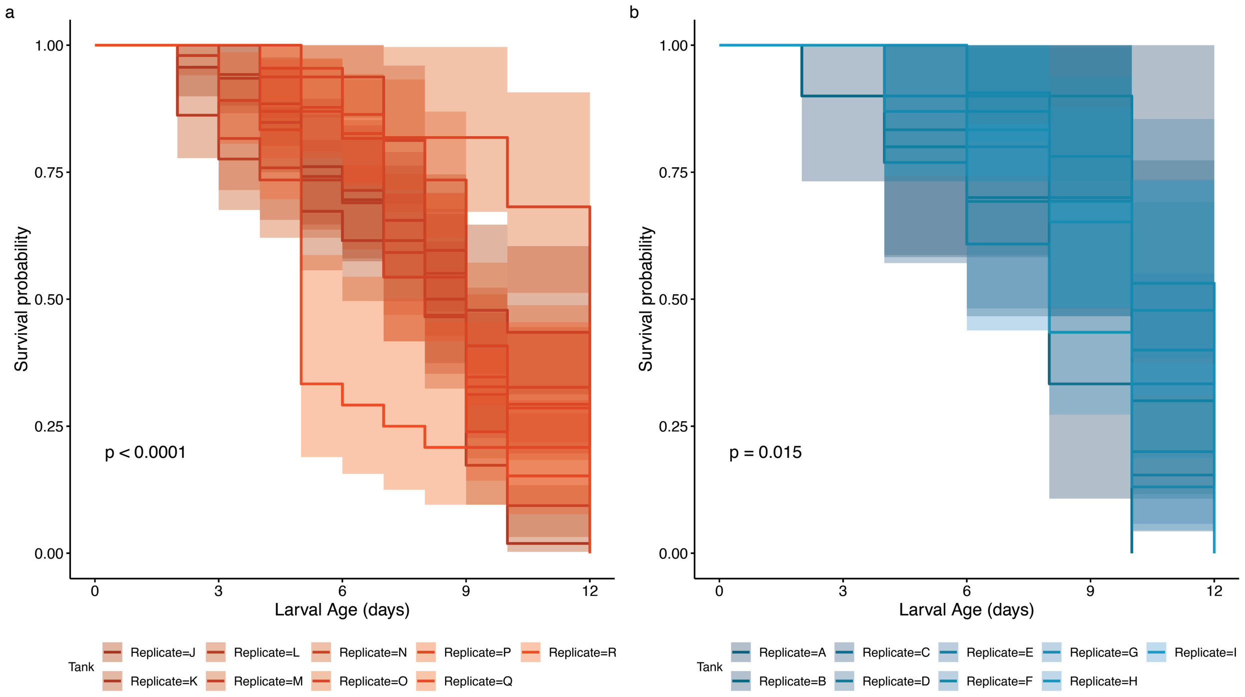

Figure S5 – Kaplan-Meier curves for each flask, separated by (a) advanced warming and (b) natural conditions.

Table S1 – Model coefficents for settlement probability. Median represents the median of the posterior distribution. CI low and high are the lower and higher values of the 95% credible interval. Rhat is the ratio of the effective sample size to the overall number of iterations, with values close to one indicating convergence values.

Parameter	Median	CI low	CI high	Rhat
Intercept	-2.66	-3.74	-1.39	1.00
Days	0.23	0.19	0.28	1.00
Treatment	-0.92	-2.27	0.60	1.00
Days:Treatment	-0.05	-0.11	0.01	1.00

Table S2 – Model coefficents for settlement rates. Median represents the median of the posterior distribution. CI low and high are the lower and higher values of the 95% credible interval. Rhat is the ratio of the effective sample size to the overall number of iterations, with values close to one indicating convergence values.

Parameter	Median	CI low	CI high	Rhat
Intercept	0.07	-0.80	0.84	1.00

Days	0.05	0.02	0.08	1.00
Treatment	-0.91	-1.93	0.21	1.00
Days:Treatment	-0.01	-0.05	0.03	1.00

Table S3 – Model coefficents for metamorphosis probability. Median represents the median of the posterior distribution. CI low and high are the lower and higher values of the 95% credible interval. Rhat is the ratio of the effective sample size to the overall number of iterations, with values close to one indicating convergence values.

Parameter	Median	CI low	CI high	Rhat
Intercept	-3.50	-5.64	-0.56	1.00
Days	0.22	0.17	0.27	1.00
Treatment	-0.69	-2.50	1.29	1.00
Days:Treatment	0.02	-0.07	0.12	1.00

Table S4 – Model coefficents for metamorphosis rates. Median represents the median of the posterior distribution. CI low and high are the lower and higher values of the 95% credible interval. Rhat is the ratio of the effective sample size to the overall number of iterations, with values close to one indicating convergence values.

Parameter	Median	CI low	CI high	Rhat
Intercept	-3.50	-5.64	-0.56	1.00
Days	0.22	0.17	0.27	1.00
Treatment	-0.69	-2.50	1.29	1.00
Days:Treatment	0.02	-0.07	0.12	1.00

Table S5 – Maximum number of dead larvae (n) quantified in the survival analysis at each tank and treatment.

Treatment	Tank	n
Advanced warming	J	46
Advanced warming	K	52
Advanced warming	L	58
Advanced warming	M	32
Advanced warming	N	49
Advanced warming	O	22
Advanced warming	P	49
Advanced warming	Q	46
Advanced warming	R	24
Natural	A	6
Natural	B	10
Natural	C	10
Natural	D	13
Natural	E	10
Natural	F	10
Natural	G	32
Natural	H	23
Natural	I	23

Table S6 – Number of settlers (n) at each tank, treatment and day.

Treatment	Tank	Day	n
Advanced warming	10	1	0
Advanced warming	10	2	0
Advanced warming	10	3	0
Advanced warming	10	4	0
Advanced warming	10	5	0
Advanced warming	10	6	0
Advanced warming	10	7	0
Advanced warming	10	8	0
Advanced warming	10	9	0
Advanced warming	10	10	0
Advanced warming	10	12	0
Advanced warming	10	14	0
Advanced warming	10	16	0
Advanced warming	10	18	3

Advanced warming	10	20	3
Advanced warming	10	22	0
Advanced warming	10	24	0
Advanced warming	10	26	0
Advanced warming	10	28	0
Advanced warming	10	30	1
Advanced warming	10	32	1
Advanced warming	10	34	0
Advanced warming	10	36	0
Advanced warming	10	38	0
Advanced warming	10	40	0
Advanced warming	10	42	0
Advanced warming	10	44	0
Advanced warming	10	46	0
Advanced warming	10	48	0
Advanced warming	10	50	0
Advanced warming	11	1	0
Advanced warming	11	2	0
Advanced warming	11	3	3
Advanced warming	11	4	3
Advanced warming	11	5	2
Advanced warming	11	6	2
Advanced warming	11	7	2
Advanced warming	11	8	2
Advanced warming	11	9	2
Advanced warming	11	10	2
Advanced warming	11	12	2
Advanced warming	11	14	2
Advanced warming	11	16	2
Advanced warming	11	18	3
Advanced warming	11	20	3
Advanced warming	11	22	2
Advanced warming	11	24	2
Advanced warming	11	26	3
Advanced warming	11	28	2
Advanced warming	11	30	2
Advanced warming	11	32	2
Advanced warming	11	34	2
Advanced warming	11	36	2

Advanced warming	11	38	2
Advanced warming	11	40	1
Advanced warming	11	42	0
Advanced warming	11	44	0
Advanced warming	11	46	0
Advanced warming	11	48	0
Advanced warming	11	50	0
Advanced warming	12	1	0
Advanced warming	12	2	1
Advanced warming	12	3	3
Advanced warming	12	4	2
Advanced warming	12	5	1
Advanced warming	12	6	1
Advanced warming	12	7	1
Advanced warming	12	8	1
Advanced warming	12	9	1
Advanced warming	12	10	1
Advanced warming	12	12	1
Advanced warming	12	14	1
Advanced warming	12	16	1
Advanced warming	12	18	1
Advanced warming	12	20	1
Advanced warming	12	22	1
Advanced warming	12	24	2
Advanced warming	12	26	3
Advanced warming	12	28	3
Advanced warming	12	30	5
Advanced warming	12	32	3
Advanced warming	12	34	1
Advanced warming	12	36	1
Advanced warming	12	38	1
Advanced warming	12	40	2
Advanced warming	12	42	2
Advanced warming	12	44	2
Advanced warming	12	46	2
Advanced warming	12	48	2
Advanced warming	12	50	2
Advanced warming	13	1	0
Advanced warming	13	2	0

Advanced warming	13	3	0
Advanced warming	13	4	0
Advanced warming	13	5	0
Advanced warming	13	6	0
Advanced warming	13	7	0
Advanced warming	13	8	0
Advanced warming	13	9	0
Advanced warming	13	10	0
Advanced warming	13	12	0
Advanced warming	13	14	0
Advanced warming	13	16	0
Advanced warming	13	18	0
Advanced warming	13	20	0
Advanced warming	13	22	0
Advanced warming	13	24	2
Advanced warming	13	26	2
Advanced warming	13	28	3
Advanced warming	13	30	2
Advanced warming	13	32	2
Advanced warming	13	34	2
Advanced warming	13	36	2
Advanced warming	13	38	2
Advanced warming	13	40	2
Advanced warming	13	42	2
Advanced warming	13	44	2
Advanced warming	13	46	2
Advanced warming	13	48	2
Advanced warming	13	50	2
Advanced warming	14	1	0
Advanced warming	14	2	0
Advanced warming	14	3	0
Advanced warming	14	4	0
Advanced warming	14	5	0
Advanced warming	14	6	0
Advanced warming	14	7	0
Advanced warming	14	8	0
Advanced warming	14	9	0
Advanced warming	14	10	0
Advanced warming	14	12	0

Advanced warming	14	14	0
Advanced warming	14	16	0
Advanced warming	14	18	0
Advanced warming	14	20	0
Advanced warming	14	22	0
Advanced warming	14	24	0
Advanced warming	14	26	0
Advanced warming	14	28	3
Advanced warming	14	30	5
Advanced warming	14	32	4
Advanced warming	14	34	3
Advanced warming	14	36	3
Advanced warming	14	38	4
Advanced warming	14	40	2
Advanced warming	14	42	2
Advanced warming	14	44	2
Advanced warming	14	46	2
Advanced warming	14	48	2
Advanced warming	14	50	2
Advanced warming	15	1	0
Advanced warming	15	2	0
Advanced warming	15	3	0
Advanced warming	15	4	0
Advanced warming	15	5	0
Advanced warming	15	6	0
Advanced warming	15	7	0
Advanced warming	15	8	0
Advanced warming	15	9	0
Advanced warming	15	10	0
Advanced warming	15	12	0
Advanced warming	15	14	0
Advanced warming	15	16	0
Advanced warming	15	18	0
Advanced warming	15	20	0
Advanced warming	15	22	0
Advanced warming	15	24	0
Advanced warming	15	26	0
Advanced warming	15	28	1
Advanced warming	15	30	0

Advanced warming	15	32	0
Advanced warming	15	34	0
Advanced warming	15	36	0
Advanced warming	15	38	0
Advanced warming	15	40	0
Advanced warming	15	42	0
Advanced warming	15	44	0
Advanced warming	15	46	0
Advanced warming	15	48	0
Advanced warming	15	50	0
Advanced warming	16	1	0
Advanced warming	16	2	0
Advanced warming	16	3	0
Advanced warming	16	4	0
Advanced warming	16	5	0
Advanced warming	16	6	0
Advanced warming	16	7	0
Advanced warming	16	8	1
Advanced warming	16	9	1
Advanced warming	16	10	1
Advanced warming	16	12	1
Advanced warming	16	14	1
Advanced warming	16	16	1
Advanced warming	16	18	1
Advanced warming	16	20	1
Advanced warming	16	22	1
Advanced warming	16	24	1
Advanced warming	16	26	2
Advanced warming	16	28	2
Advanced warming	16	30	2
Advanced warming	16	32	2
Advanced warming	16	34	2
Advanced warming	16	36	2
Advanced warming	16	38	2
Advanced warming	16	40	1
Advanced warming	16	42	1
Advanced warming	16	44	1
Advanced warming	16	46	1
Advanced warming	16	48	1

Advanced warming	16	50	1
Advanced warming	18	1	0
Advanced warming	18	2	0
Advanced warming	18	3	0
Advanced warming	18	4	0
Advanced warming	18	5	0
Advanced warming	18	6	0
Advanced warming	18	7	0
Advanced warming	18	8	0
Advanced warming	18	9	0
Advanced warming	18	10	0
Advanced warming	18	12	1
Advanced warming	18	14	1
Advanced warming	18	16	1
Advanced warming	18	18	1
Advanced warming	18	20	3
Advanced warming	18	22	1
Advanced warming	18	24	7
Advanced warming	18	26	7
Advanced warming	18	28	6
Advanced warming	18	30	2
Advanced warming	18	32	2
Advanced warming	18	34	2
Advanced warming	18	36	1
Advanced warming	18	38	1
Advanced warming	18	40	1
Advanced warming	18	42	2
Advanced warming	18	44	2
Advanced warming	18	46	2
Advanced warming	18	48	2
Advanced warming	18	50	2
Natural	1	2	0
Natural	1	4	0
Natural	1	6	0
Natural	1	8	0
Natural	1	10	0
Natural	1	12	1
Natural	1	14	4
Natural	1	16	9

Natural	1	18	17
Natural	1	20	15
Natural	1	22	29
Natural	1	24	27
Natural	1	26	26
Natural	1	28	28
Natural	1	30	33
Natural	1	32	34
Natural	1	34	29
Natural	1	36	29
Natural	1	38	33
Natural	1	40	33
Natural	1	42	21
Natural	1	44	12
Natural	1	46	11
Natural	1	48	11
Natural	1	50	9
Natural	2	2	0
Natural	2	4	0
Natural	2	6	0
Natural	2	8	0
Natural	2	10	0
Natural	2	12	3
Natural	2	14	6
Natural	2	16	19
Natural	2	18	31
Natural	2	20	32
Natural	2	22	34
Natural	2	24	36
Natural	2	26	29
Natural	2	28	25
Natural	2	30	28
Natural	2	32	26
Natural	2	34	20
Natural	2	36	21
Natural	2	38	22
Natural	2	40	20
Natural	2	42	15
Natural	2	44	14

Natural	2	46	13
Natural	2	48	12
Natural	2	50	12
Natural	3	2	0
Natural	3	4	0
Natural	3	6	0
Natural	3	8	0
Natural	3	10	0
Natural	3	12	0
Natural	3	14	0
Natural	3	16	0
Natural	3	18	0
Natural	3	20	2
Natural	3	22	3
Natural	3	24	6
Natural	3	26	4
Natural	3	28	6
Natural	3	30	8
Natural	3	32	8
Natural	3	34	7
Natural	3	36	9
Natural	3	38	11
Natural	3	40	12
Natural	3	42	6
Natural	3	44	2
Natural	3	46	2
Natural	3	48	2
Natural	3	50	3
Natural	4	2	0
Natural	4	4	0
Natural	4	6	0
Natural	4	8	0
Natural	4	10	2
Natural	4	12	0
Natural	4	14	0
Natural	4	16	4
Natural	4	18	6
Natural	4	20	5
Natural	4	22	3

Natural	4	24	3
Natural	4	26	3
Natural	4	28	3
Natural	4	30	3
Natural	4	32	3
Natural	4	34	3
Natural	4	36	3
Natural	4	38	3
Natural	4	40	3
Natural	4	42	2
Natural	4	44	2
Natural	4	46	2
Natural	4	48	2
Natural	4	50	2
Natural	5	2	0
Natural	5	4	0
Natural	5	6	0
Natural	5	8	0
Natural	5	10	3
Natural	5	12	2
Natural	5	14	3
Natural	5	16	2
Natural	5	18	4
Natural	5	20	5
Natural	5	22	3
Natural	5	24	1
Natural	5	26	2
Natural	5	28	2
Natural	5	30	2
Natural	5	32	2
Natural	5	34	2
Natural	5	36	2
Natural	5	38	2
Natural	5	40	2
Natural	5	42	2
Natural	5	44	2
Natural	5	46	2
Natural	5	48	2
Natural	5	50	2

Natural	6	2	0
Natural	6	4	0
Natural	6	6	0
Natural	6	8	0
Natural	6	10	0
Natural	6	12	1
Natural	6	14	0
Natural	6	16	0
Natural	6	18	0
Natural	6	20	1
Natural	6	22	1
Natural	6	24	1
Natural	6	26	1
Natural	6	28	1
Natural	6	30	1
Natural	6	32	1
Natural	6	34	1
Natural	6	36	1
Natural	6	38	1
Natural	6	40	1
Natural	6	42	1
Natural	6	44	1
Natural	6	46	1
Natural	6	48	1
Natural	6	50	1
Natural	8	2	0
Natural	8	4	0
Natural	8	6	1
Natural	8	8	1
Natural	8	10	1
Natural	8	12	1
Natural	8	14	1
Natural	8	16	2
Natural	8	18	2
Natural	8	20	9
Natural	8	22	12
Natural	8	24	10
Natural	8	26	11
Natural	8	28	8

Natural	8	30	1
Natural	8	32	1
Natural	8	34	1
Natural	8	36	1
Natural	8	38	1
Natural	8	40	1
Natural	8	42	1
Natural	8	44	1
Natural	8	46	0
Natural	8	48	0
Natural	8	50	0
Natural	9	2	0
Natural	9	4	1
Natural	9	6	1
Natural	9	8	4
Natural	9	10	7
Natural	9	12	6
Natural	9	14	11
Natural	9	16	17
Natural	9	18	18
Natural	9	20	21
Natural	9	22	17
Natural	9	24	17
Natural	9	26	13
Natural	9	28	9
Natural	9	30	9
Natural	9	32	9
Natural	9	34	8
Natural	9	36	7
Natural	9	38	4
Natural	9	40	3
Natural	9	42	3
Natural	9	44	3
Natural	9	46	3
Natural	9	48	3
Natural	9	50	3

Table S7 – Number of initial larvae and metamorphosed larvae, per day, tank and treatment.

Treatment	Tank	Day	Larvae	Metamorphosed
Natural	A	0	300	0
Natural	A	2	300	0
Natural	A	4	299	0
Natural	A	6	299	0
Natural	A	8	296	0
Natural	A	10	296	0
Natural	A	12	294	0
Natural	A	14	290	0
Natural	A	16	281	0
Natural	A	18	254	0
Natural	A	20	227	4
Natural	A	22	189	6
Natural	A	24	159	6
Natural	A	26	126	6
Natural	A	28	90	6
Natural	A	30	59	7
Natural	A	32	43	8
Natural	A	34	36	8
Natural	A	36	33	10
Natural	A	38	28	8
Natural	A	40	25	10
Natural	A	42	19	5
Natural	A	44	3	18
Natural	A	46	3	18
Natural	A	48	2	17
Natural	A	50	1	17
Natural	B	0	300	0
Natural	B	2	299	0
Natural	B	4	298	0
Natural	B	6	297	0
Natural	B	8	297	0
Natural	B	10	293	0
Natural	B	12	290	0
Natural	B	14	280	0
Natural	B	16	260	0

Natural	B	18	224	0
Natural	B	20	156	2
Natural	B	22	102	1
Natural	B	24	68	0
Natural	B	26	53	0
Natural	B	28	31	0
Natural	B	30	21	0
Natural	B	32	21	1
Natural	B	34	20	0
Natural	B	36	18	0
Natural	B	38	15	0
Natural	B	40	15	2
Natural	B	42	15	0
Natural	B	44	15	1
Natural	B	46	12	2
Natural	B	48	8	3
Natural	B	50	8	2
Natural	C	0	300	0
Natural	C	2	300	0
Natural	C	4	300	0
Natural	C	6	298	0
Natural	C	8	297	0
Natural	C	10	290	0
Natural	C	12	290	0
Natural	C	14	239	0
Natural	C	16	196	0
Natural	C	18	190	0
Natural	C	20	184	0
Natural	C	22	174	0
Natural	C	24	149	2
Natural	C	26	137	1
Natural	C	28	104	2
Natural	C	30	89	4
Natural	C	32	78	2
Natural	C	34	71	2
Natural	C	36	59	4
Natural	C	38	51	7
Natural	C	40	43	9
Natural	C	42	38	9

Natural	C	44	27	9
Natural	C	46	27	9
Natural	C	48	23	11
Natural	C	50	22	10
Natural	D	0	300	0
Natural	D	2	300	0
Natural	D	4	297	0
Natural	D	6	296	0
Natural	D	8	294	0
Natural	D	10	289	0
Natural	D	12	287	0
Natural	D	14	268	0
Natural	D	16	164	0
Natural	D	18	46	0
Natural	D	20	7	0
Natural	D	22	3	0
Natural	D	24	3	0
Natural	D	26	2	0
Natural	D	28	2	0
Natural	D	30	2	0
Natural	D	32	2	0
Natural	D	34	2	0
Natural	D	36	0	0
Natural	D	38	0	0
Natural	D	40	0	0
Natural	D	42	0	0
Natural	D	44	0	0
Natural	D	46	0	0
Natural	D	48	0	0
Natural	D	50	0	0
Natural	E	0	300	0
Natural	E	2	300	0
Natural	E	4	299	0
Natural	E	6	299	0
Natural	E	8	297	0
Natural	E	10	292	0
Natural	E	12	290	0
Natural	E	14	265	0
Natural	E	16	167	0

Natural	E	18	18	0
Natural	E	20	6	0
Natural	E	22	2	0
Natural	E	24	2	0
Natural	E	26	0	0
Natural	E	28	0	0
Natural	E	30	0	0
Natural	E	32	0	0
Natural	E	34	0	0
Natural	E	36	0	0
Natural	E	38	0	0
Natural	E	40	0	0
Natural	E	42	0	0
Natural	E	44	0	0
Natural	E	46	0	0
Natural	E	48	0	0
Natural	E	50	0	0
Natural	F	0	300	0
Natural	F	2	300	0
Natural	F	4	299	0
Natural	F	6	299	0
Natural	F	8	299	0
Natural	F	10	294	0
Natural	F	12	290	0
Natural	F	14	283	0
Natural	F	16	234	0
Natural	F	18	63	0
Natural	F	20	14	0
Natural	F	22	6	0
Natural	F	24	3	0
Natural	F	26	3	0
Natural	F	28	1	0
Natural	F	30	1	0
Natural	F	32	1	0
Natural	F	34	1	0
Natural	F	36	1	0
Natural	F	38	1	0
Natural	F	40	1	0
Natural	F	42	1	0

Natural	F	44	1	0
Natural	F	46	1	0
Natural	F	48	1	0
Natural	F	50	1	0
Natural	H	0	300	0
Natural	H	2	300	0
Natural	H	4	300	0
Natural	H	6	297	0
Natural	H	8	292	0
Natural	H	10	288	0
Natural	H	12	277	0
Natural	H	14	255	0
Natural	H	16	204	1
Natural	H	18	119	6
Natural	H	20	55	3
Natural	H	22	8	0
Natural	H	24	6	0
Natural	H	26	5	0
Natural	H	28	2	0
Natural	H	30	0	0
Natural	H	32	0	0
Natural	H	34	0	0
Natural	H	36	0	0
Natural	H	38	0	0
Natural	H	40	0	0
Natural	H	42	0	0
Natural	H	44	0	0
Natural	H	46	0	1
Natural	H	48	0	1
Natural	H	50	0	1
Natural	I	0	300	0
Natural	I	2	300	0
Natural	I	4	297	0
Natural	I	6	291	0
Natural	I	8	287	0
Natural	I	10	280	0
Natural	I	12	277	1
Natural	I	14	257	3
Natural	I	16	233	2

Natural	I	18	187	6
Natural	I	20	68	3
Natural	I	22	13	0
Natural	I	24	10	2
Natural	I	26	7	0
Natural	I	28	3	0
Natural	I	30	3	0
Natural	I	32	2	0
Natural	I	34	2	0
Natural	I	36	2	0
Natural	I	38	2	0
Natural	I	40	2	0
Natural	I	42	2	0
Natural	I	44	1	0
Natural	I	46	1	0
Natural	I	48	1	0
Natural	I	50	1	0
Advanced warming	J	0	300	0
Advanced warming	J	2	298	0
Advanced warming	J	4	293	0
Advanced warming	J	6	286	0
Advanced warming	J	8	277	0
Advanced warming	J	10	274	0
Advanced warming	J	12	254	0
Advanced warming	J	14	246	0
Advanced warming	J	16	240	0
Advanced warming	J	18	209	0
Advanced warming	J	20	204	0
Advanced warming	J	22	170	0

Advanced warming	J	24	108	0
Advanced warming	J	26	61	0
Advanced warming	J	28	12	0
Advanced warming	J	30	4	0
Advanced warming	J	32	2	0
Advanced warming	J	34	1	0
Advanced warming	J	36	1	0
Advanced warming	J	38	1	0
Advanced warming	J	40	1	0
Advanced warming	J	42	1	0
Advanced warming	J	44	1	0
Advanced warming	J	46	1	0
Advanced warming	J	48	0	0
Advanced warming	J	50	0	0
Advanced warming	K	0	300	0
Advanced warming	K	2	300	0
Advanced warming	K	4	294	0
Advanced warming	K	6	280	0
Advanced warming	K	8	279	0
Advanced warming	K	10	249	0
Advanced warming	K	12	248	0

Advanced warming	K	14	240	0
Advanced warming	K	16	235	0
Advanced warming	K	18	222	0
Advanced warming	K	20	221	0
Advanced warming	K	22	220	0
Advanced warming	K	24	199	0
Advanced warming	K	26	180	0
Advanced warming	K	28	143	0
Advanced warming	K	30	103	0
Advanced warming	K	32	62	1
Advanced warming	K	34	38	1
Advanced warming	K	36	14	0
Advanced warming	K	38	4	0
Advanced warming	K	40	3	0
Advanced warming	K	42	3	0
Advanced warming	K	44	3	0
Advanced warming	K	46	3	0
Advanced warming	K	48	3	0
Advanced warming	K	50	3	0
Advanced warming	L	0	300	0
Advanced warming	L	2	292	0

Advanced warming	L	4	286	0
Advanced warming	L	6	282	0
Advanced warming	L	8	269	0
Advanced warming	L	10	259	0
Advanced warming	L	12	242	0
Advanced warming	L	14	237	0
Advanced warming	L	16	223	0
Advanced warming	L	18	214	0
Advanced warming	L	20	202	0
Advanced warming	L	22	182	0
Advanced warming	L	24	153	0
Advanced warming	L	26	97	0
Advanced warming	L	28	69	0
Advanced warming	L	30	24	0
Advanced warming	L	32	8	0
Advanced warming	L	34	4	0
Advanced warming	L	36	2	0
Advanced warming	L	38	2	0
Advanced warming	L	40	1	0
Advanced warming	L	42	1	0
Advanced warming	L	44	1	0

Advanced warming	L	46	1	0
Advanced warming	L	48	1	0
Advanced warming	L	50	1	0
Advanced warming	M	0	300	0
Advanced warming	M	2	300	0
Advanced warming	M	4	298	0
Advanced warming	M	6	298	0
Advanced warming	M	8	283	0
Advanced warming	M	10	271	0
Advanced warming	M	12	268	0
Advanced warming	M	14	256	0
Advanced warming	M	16	250	0
Advanced warming	M	18	246	0
Advanced warming	M	20	240	0
Advanced warming	M	22	219	0
Advanced warming	M	24	168	0
Advanced warming	M	26	93	0
Advanced warming	M	28	58	0
Advanced warming	M	30	34	0
Advanced warming	M	32	14	0
Advanced warming	M	34	4	0

Advanced warming	M	36	2	0
Advanced warming	M	38	2	0
Advanced warming	M	40	0	0
Advanced warming	M	42	0	0
Advanced warming	M	44	0	0
Advanced warming	M	46	0	0
Advanced warming	M	48	0	0
Advanced warming	M	50	0	0
Advanced warming	N	0	261	0
Advanced warming	N	2	260	0
Advanced warming	N	4	248	0
Advanced warming	N	6	247	0
Advanced warming	N	8	239	0
Advanced warming	N	10	226	0
Advanced warming	N	12	212	0
Advanced warming	N	14	204	0
Advanced warming	N	16	194	0
Advanced warming	N	18	193	0
Advanced warming	N	20	189	0
Advanced warming	N	22	163	0
Advanced warming	N	24	62	0

Advanced warming	N	26	28	0
Advanced warming	N	28	14	0
Advanced warming	N	30	12	1
Advanced warming	N	32	12	0
Advanced warming	N	34	11	0
Advanced warming	N	36	10	0
Advanced warming	N	38	7	1
Advanced warming	N	40	7	0
Advanced warming	N	42	7	0
Advanced warming	N	44	7	0
Advanced warming	N	46	5	0
Advanced warming	N	48	3	2
Advanced warming	N	50	3	2
Advanced warming	O	0	300	0
Advanced warming	O	2	300	0
Advanced warming	O	4	299	0
Advanced warming	O	6	297	0
Advanced warming	O	8	296	0
Advanced warming	O	10	293	0
Advanced warming	O	12	278	0
Advanced warming	O	14	267	0

Advanced warming	O	16	264	0
Advanced warming	O	18	263	0
Advanced warming	O	20	254	0
Advanced warming	O	22	211	0
Advanced warming	O	24	91	0
Advanced warming	O	26	13	0
Advanced warming	O	28	5	0
Advanced warming	O	30	4	0
Advanced warming	O	32	3	0
Advanced warming	O	34	3	0
Advanced warming	O	36	3	0
Advanced warming	O	38	2	0
Advanced warming	O	40	2	0
Advanced warming	O	42	2	0
Advanced warming	O	44	2	0
Advanced warming	O	46	2	0
Advanced warming	O	48	2	0
Advanced warming	O	50	1	0
Advanced warming	P	0	300	0
Advanced warming	P	2	300	0
Advanced warming	P	4	300	0

Advanced warming	P	6	291	0
Advanced warming	P	8	287	0
Advanced warming	P	10	267	0
Advanced warming	P	12	251	0
Advanced warming	P	14	247	0
Advanced warming	P	16	237	0
Advanced warming	P	18	224	0
Advanced warming	P	20	109	0
Advanced warming	P	22	58	0
Advanced warming	P	24	33	0
Advanced warming	P	26	17	0
Advanced warming	P	28	15	0
Advanced warming	P	30	6	0
Advanced warming	P	32	5	0
Advanced warming	P	34	4	0
Advanced warming	P	36	4	0
Advanced warming	P	38	3	0
Advanced warming	P	40	3	0
Advanced warming	P	42	2	1
Advanced warming	P	44	2	1
Advanced warming	P	46	2	1

Advanced warming	P	48	2	1
Advanced warming	P	50	1	2
Advanced warming	R	0	300	0
Advanced warming	R	2	300	0
Advanced warming	R	4	296	0
Advanced warming	R	6	283	0
Advanced warming	R	8	281	0
Advanced warming	R	10	281	0
Advanced warming	R	12	276	0
Advanced warming	R	14	273	0
Advanced warming	R	16	272	0
Advanced warming	R	18	269	0
Advanced warming	R	20	177	0
Advanced warming	R	22	99	0
Advanced warming	R	24	49	0
Advanced warming	R	26	29	0
Advanced warming	R	28	21	0
Advanced warming	R	30	14	0
Advanced warming	R	32	8	0
Advanced warming	R	34	7	0
Advanced warming	R	36	6	0

Advanced warming	R	38	6	0
Advanced warming	R	40	5	0
Advanced warming	R	42	4	0
Advanced warming	R	44	4	0
Advanced warming	R	46	4	0
Advanced warming	R	48	4	0
Advanced warming	R	50	3	1
